# Bupropion-Induced Acute Dystonia with Dose Escalation and Use of Naranjo Nomogram

**DOI:** 10.7759/cureus.1157

**Published:** 2017-04-12

**Authors:** Nawal Wasif, Komal Wasif, Muhammad W Saif

**Affiliations:** 1 Tufts Medical Center, Tufts University School of Medicine; 2 Brookline High School, Tufts Medical Center; 3 Hematology/Oncology, Tufts Medical Center, Tufts University School of Medicine

**Keywords:** depression, smoking cessation, dystonia, antidepressants, neck stiffness, trismus, temporomandibular joint (tmj), tardive dystonia, tardive dyskinesias

## Abstract

Acute drug-induced dystonia is commonly associated with antipsychotic drugs, antidepressants, antiemetics, and other medications. Bupropion (Wellbutrin and Zyban) is one of the most frequently prescribed antidepressants in the United States and Canada and smoking cessation aid. However, only few reported cases have been published of acute dystonia including dystonia after discontinuation of bupropion and even after a single dose of bupropion. Here, we report another case concerning an acute dystonia resulting from bupropion after dose escalation. To further assess this association, we used the Naranjo nomogram, which is a questionnaire designed for determining the likelihood of whether an adverse drug reaction is actually due to the drug rather than the result of other factors. Our patient’s total score was seven, suggesting that our patient had probable adverse drug reaction. In summary, our case is that selected patients may experience dose-related acute dystonia as adverse reactions to bupropion sustained release (SR). Since it is one of the most commonly prescribed antidepressants and smoking cessation aids, clinicians should be aware of the potential dystonia associated with bupropion.

## Introduction

Bupropion (marketed as Wellbutrin and Zyban) belongs to the class of aminoketones and is a norepinephrine-dopamine reuptake inhibitor (NDRI) and a nicotinic antagonist. It is primarily prescribed as an antidepressant to treat major depressive disorders, sexual side effects of selective serotonin reuptake inhibitors, and as a smoking cessation aid [1-2]. Common adverse effects include dry mouth, nausea, insomnia, headache, anxiety, tremor, dizziness, and constipation [1-2] (Table 1).

**Table 1:** List of side effects of bupropion

>10%	1–10%	< 1%	0.01–0.1%
Headache	Agitation or Anxiety	Anorexia	Seizures
Transient insomnia	Asthenia	Confusion	Ataxia
	Dry mouth	Flushing	Delusions
	Taste disorders	Hypertension, tachycardia, chest pain	Depersonalization
	Tremor	Tinnitus	Hallucinations
	Rash	>Alopecia	Hostility, aggression, irritability
	Visual disturbance		Memory impairment
	Concentration disturbance		Paraesthesia
	Constipation, vomiting, abdominal pain		>Anaphylactic shock Angioedema
	Dizziness		Angioedema
	Sweating		Twitching
	Fever		Urinary retention
	Depression		>Orthostatic hypotension
			Parkinsonism
			Paranoid ideation
			Abnormal dreams

The drug label cautions that bupropion should not be prescribed to individuals with history of epilepsy or any other conditions that may decrease the threshold for seizure including anorexia nervosa, bulimia nervosa, active brain tumors, or concurrent alcohol and/or benzodiazepine use and/or withdrawal. However, neurological toxicities such as dystonia are only rarely publshed. There are several case reports about acute dystonia resulting from bupropion [3-7]. Acute dystonia is common with first-generation antipsychotics with a high potency.

## Case presentation

The patient was a 47-year-old Asian man with a history of borderline diabetes taking metformin, ramipril, and Celexa 20 mg (citalopram hydrobromide) for chronic depression. Due to recent divorce issues, his depression worsened and his dose of Celexa was increased to 40 mg with no improvement. Therefore, he was started on bupropion SR (sustained release) 150 mg once a day for depression, which he tolerated very well. The dosage was increased to 300 mg SR once a day on the fourth day if tolerated. On day 9, he developed severe neck stiffness, intense neck involuntary movements towards the left side with a frequency of 20 per hour making him unable to work on a computer or drive, inability to rotate his head laterally, and spontaneous left tempromandibular joint (TMJ) subluxation. There were no associated signs and symproms such as fever, head trauma, seizure, or substance abuse. There was no family history of head trauma, seizure or substance abuse, or family history of psychiatric disorders and abnormal movement disorders. He had no relevant past psychiatric history, conversion nor malingering.

The Abnormal Involuntary Movement Scale (AIMS) score is a rating scale that is usually used to measure involuntary movements known as tardive dyskinesia (TD), a syndrome characterized by abnormal involuntary movements of the patient's face, mouth, trunk, or limbs [8]. TD affects 20%–30% of patients who have received neuroleptic/antipsychotic medications.​ The AIMS score in our patient was > 2 based on moderate movements in one area. The rest of the neurological examination was normal. Complete blood count, chemistry, serum calcium/magnesium, vitamin B12 level, folic acid level, ferritin, and a computerized tomography (CT) scan of neck were perfomed, and no abnormalities were found. In addition, screening for Wilson's disease was undertaken with no abnormality found. Hence, the diagnosis of secondary acute dystonia diagnosis was connected to bupropion.

Bupropion SR was halted, and he was prescribed diazepam 5 mg once a day. His dystonic symptoms recessed with discontinuation of bupropion SR after a week. He was offered to re-challenge with a lower dose but he declined. He was therefore switched back to Celexa 40 mg once a day and those symptoms did not recur. At follow-ups two weeks, four weeks, six weeks, and 12 weeks later, no recurrence of symptoms of dystonia occurred. His depression remained stable with mild improvement.

To further assess this association, we used the Naranjo algorithm to further determine the likelihood of dystonia as an adverse drug reaction due to bupropion SR (Table 2) [9].

**Table 2:** The Naranjo probability scale

Item	Score	Our patient
1. Are there previous conclusive reports on this reaction?	Yes (+1); No (0); Do not know or not done (0)	+1
2. Did the adverse event appear after the suspected drug was given?	Yes (+2); No (-1); Do not know or not done (0)	+2
3. Did the adverse reaction improve when the drug was discontinued or a specific antagonist was given?	Yes (+1); No (0); Do not know or not done (0)	+1
4. Did the adverse reaction appear when the drug was readministered?	Yes (+2); No (-2); Do not know or not done (0)	0
5. Are there alternative causes that could have caused the reaction?	Yes (-1); No (+2); Do not know or not done (0)	+2
6. Did the reaction reappear when a placebo was given?	Yes (-1); No (+1); Do not know or not done (0)	0
7. Was the drug detected in any body fluid in toxic concentrations?	Yes (+1); No (0); Do not know or not done (0)	0
8. Was the reaction more severe when the dose was increased, or less severe when the dose was decreased?	Yes (+1); No (0); Do not know or not done (0)	+1
9. Did the patient have a similar reaction to the same or similar drugs in any previous exposure?	Yes (+1); No (0); Do not know or not done (0)	0
10. Was the adverse event confirmed by any objective evidence?	Yes (+1); No (0); Do not know or not done (0)	0

Our patient’s total score was seven. Based on the Naranjo algorithm, our patient had probable adverse drug reaction.

## Discussion

Medication-induced focal dystonias usually present with dramatic and twisting involuntary movements caused by head and neck muscle spasm and are sometimes also associated with jaw clenching, bruxism, and TMJ syndrome. Acute dystonia can occur several hours to several days after beginning, increasing, or decreasing drug dose.

This case reports that acute dystonia due to bupropion SR occurred when an escalated dose from 150 to 300 mg daily was administered. We reviewed the medical literature and found only limited data consisting of scattered cases, including a report of acute dystonia after discontinuation of bupropion; one suffered from acute dystonia symptoms after a single dose of bupropion [3-7]. These symptoms may begin to appear within hours of starting or dose escalation of the drug. Literature review also underlines the fact that concurrent use of bupropion with a serotonin reuptake agent such as buspirone or an selective serotonin reuptake inhibitors (SSRIs) may cause acute dystonia. In addition to this, concurrent use of St. John's wort with bupropion in another patient resulted in dystonia. It is well known that dystonia is a side effect of several antipsychotics, antidepressants especially SSRIs, antiemetics, and other medications (Table 3).

**Table 3:** Most common drugs known to cause dystonia

Drugs known to cause dystonia
Acetohenazine
Amoxapine
Chlorpromazine
Fluphenazine
Haloperidol
Loxapine
Mesoridazine
Metaclopramide
Molinndone
Perphanzine
Piperacetazine
Prochlorperzine
Promazine
Promethazine
Thiethylperazine
Thioridazine
Thiothixene
Triflupromazine
Trimeprazine

The exact underlying mechanism responsbile to cause dystonia by bupropion is suggested to be the effect of bupropion to strengthen dopaminergic and noradrenergic activity as it is both a dopamine and noradrenaline reuptake inhibitor [1-2,10].

The Abnormal Involuntary Movement Scale (AIMS) is a rating scale that was designed in the 1970s to measure involuntary movements known as tardive dyskinesia (TD) [8]. The AIMS is a 12-item anchored scale that is clinician administered, and scores in items 1-10 are rated on a five-point anchored scale. Items one to four assess orofacial movements. Items five to seven deal with extremity and truncal dyskinesia. Items 8-10 deal with global severity as judged by the examiner and the patient’s awareness of the movements and the distress associated with them. In Items 11-12 are yes-no questions concerning problems with teeth and/or dentures because such problems can lead to a mistaken diagnosis of dyskinesia. A rating of two or higher on the AIMS scale, however, is evidence of tardive dyskinesia. If the patient has mild TD in two areas or moderate movements in one area, then he or she should be given a diagnosis of TD. The AIMS test is considered an extremely reliable tool.

The Naranjo algorithm (Naranjo Scale; Naranjo Nomogram) is a questionnaire designed to determine the likelihood of whether an adverse drug reaction is actually secondary to the drug rather than the result of other factors [9]. Probability of adverse drug reaction is assigned via a score termed definite (total score greater than nine), probable (total score ranging five to eight), possible (total score ranging one to four) or doubtful (total score equal to zero).

The differential diagnosis, however, should include hypocalcemia, Wilson's disease, encephalitis, tetanus, catatonia, and conversion and malingering disorders.

In a review of the Wellbutrin XL PI (http://www.wellbutrinxl.com), it is shown that following chronic dosing, the mean steady-state plasma concentration of bupropion was reached within eight days. The mean elimination half-life (±SD) of bupropion is 21 (±9) hours. In addition, a study compared 14-day dosing with Wellbutrin XL 300 mg once-daily to the immediate-release formulation of bupropion at 100 mg three times daily and showed equivalence for both peak plasma concentration and area under the curve for bupropion and the three metabolites (hydroxybupropion, threohydrobupropion, and erythrohydrobupropion). Similarly, another study compared 14-day dosing with Wellbutrin XL 300 mg once daily to the sustained-release formulation of bupropion at 150 mg two times daily and showed equivalent results similar to the previous study.

## Conclusions

In summary, our case report reinforces the fact that acute dystonia is a potential side effect of bupropion, which is painful and terrifying. The above case also suggests that escalation of dosage might have contributed, but it is also important to remember that bupropion's mean elimination half-life (±SD) is 21 (±9) hours. The physicians caring for these patients should be aware of acute dystonia associated with bupropion, and proper management of this potentially dangerous side effect including stopping the agent or reducing the dose can be helpful. Moreover, caution must be used when prescribing bupropion concurrently with other potential culprit drugs, especially those that have the same effect on serotonin reuptake.

## References

[1] Dwoskin LP, Rauhut AS, King-Pospisil KA (2006). Review of the pharmacology and clinical profile of bupropion, an antidepressant and tobacco use cessation agent. CNS Drug Rev.

[2] Stahl SM, Pradko JF, Haight BR (2004). A review of the neuropharmacology of bupropion, a dual norepinephrine and dopamine reuptake inhibitor. Prim Care Companion J Clin Psychiatry.

[3] Detweiler MB, Harpold GJ (2002). Bupropion-induced acute dystonia. Ann Pharmacother.

[4] Wang HY, Chou WJ, Huang TY (2007). Acute dystonia resulting from abrupt bupropion discontinuation. Prog Neuropsychopharmacol Biol Psychiatry.

[5] Elyasi F, Mahtiyan E (2016). Acute dystonia after single dose of bupropion. Indian J Psychol Med.

[6] Strouse TB, Salehmoghaddam S, Spar JE (1993). Acute delirium and parkinsonism in a bupropion-treated liver transplant recipient. J Clin Psychiatry.

[7] Demir S, Atli A, Gunes M (2015). Single dose bupropion related acute dystonia: a case report. [Article in Turkish]. Anadolu Psikiyatri Derg.

[8] Munetz MR, Benjamin S (1988). How to examine patients using the Abnormal Involuntary Movement Scale. Hosp Community Psychiatry.

[9] Naranjo CA, Busto U, Sellers EM (1981). A method for estimating the probability of adverse drug reactions. Clin Pharmacol Ther.

[10] Korczyn AD (1978). The pathophysiology of dystonia. J Neural Transm.

